# Single Photon Counting Performance and Noise Analysis of CMOS SPAD-Based Image Sensors

**DOI:** 10.3390/s16071122

**Published:** 2016-07-20

**Authors:** Neale A. W. Dutton, Istvan Gyongy, Luca Parmesan, Robert K. Henderson

**Affiliations:** 1 STMicroelectronics Imaging Division, Pinkhill, Edinburgh EH12 7BF, UK; 2 CMOS Sensors and Systems Group, School of Engineering, The University of Edinburgh, Edinburgh EH9 3JL, UK; Istvan.Gyongy@ed.ac.uk (I.G.); l.parmesan@ed.ac.uk (L.P.); robert.henderson@ed.ac.uk (R.K.H.)

**Keywords:** single photon avalanche diode, SPAD, CMOS image sensor, CIS, single photon counting, SPC, quanta image sensor, QIS, spatio-temporal oversampling

## Abstract

SPAD-based solid state CMOS image sensors utilising analogue integrators have attained deep sub-electron read noise (DSERN) permitting single photon counting (SPC) imaging. A new method is proposed to determine the read noise in DSERN image sensors by evaluating the peak separation and width (PSW) of single photon peaks in a photon counting histogram (PCH). The technique is used to identify and analyse cumulative noise in analogue integrating SPC SPAD-based pixels. The DSERN of our SPAD image sensor is exploited to confirm recent multi-photon threshold quanta image sensor (QIS) theory. Finally, various single and multiple photon spatio-temporal oversampling techniques are reviewed.

## 1. Introduction

Imaging a few photons per pixel, per frame, demands pixels operating in the single photon counting regime. This challenge is encountered in either low-light or high-speed imaging; at long (ms to s) integration times and low photon flux, or short (µs or less) integration times and high photon flux, respectively. Examples are high-speed cameras for engine and exhaust combustion analysis, low-light or night-vision cameras for defence [1], staring applications in astronomy and many scientific applications such as, spectroscopy, fluorescence lifetime imaging microscopy (FLIM) [2,3], positron emission tomography (PET) [4], fluorescence correlation spectroscopy (FCS) [5], Förster Resonance Emission Tomography (FRET) [6], and in automotive applications for LIDAR [7]. 

For true photoelectron (or photon) counting to be reached, the ratio of the input sensitivity or signal to the noise of the imaging system must be sufficiently high to allow discrete and resolvable signal levels for each photoelectron to be discriminated. Referring the readout noise to the input sensitivity in photoelectrons, the single photon counting regime is theoretically entered below 0.5 e^−^ input referred read noise (RN) [8], but practically there is a 90% accuracy of determining the number of photoelectrons at 0.3 e^−^ RN, and approaching 100% accuracy at 0.15 e^−^ RN [9]. These probability figures, assume RN is Gaussian distributed and the discrimination thresholds between one photoelectron signal, to the next, are set precisely mid-way and do not take into account fixed pattern noise (FPN) or gain variations in photo-response non-uniformity (PRNU). Such sensors in this photon-counting regime with approximately <0.3 e^−^ RN may be referred to as deep sub-electron read noise (DSERN) image sensors [10].

With high charge to voltage factor (CVF) sensitivity (or conversion gain (CG)), DSERN pixels have limited photoelectron or photon counting capability (full well capacity), and therefore restricted dynamic range (DR). DR may be extended by a range of techniques: exposure control with the capture of multiple sequential images [11], pixel design with dual integrations (e.g., lateral overflow integration capacitors (LOFIC) [12]), or by combining multiple pixel samples through spatio-temporal oversampling [13,14]. In the latter the number of oversampled frames is traded off against the frame rate.

This paper evaluates the single photon counting and noise characteristics of our recent work on SPAD-based image sensors [15,16,17] and analyses the benefits, tradeoffs and noise performance of various spatio-temporal oversampling techniques [18,19]. A new method of determining RN, CVF and other imaging measurements of DSERN image sensors is described.

## 2. Solid-State Single Photon Counting Imaging Background

Since the late 1980s, single photon counting (SPC) and time-gated imaging have been dominated by photo-cathode based intensifier techniques achieving high signal amplification through the “photo-intensification” of the generated electron cascade through the photo-electric effect using existing charge-coupled device (CCD) and CMOS image sensors (CIS) [1]. However, there are a number of drawbacks which limit their usage dependent on the application. Namely, the wavelength (colour) and spin properties of the photons are lost. Systems have high cost and are physically bulky due to the requirement of operation in a vacuum. Furthermore, photo-cathodes are sensitive to magnetic fields, they have high (kV) operating voltage and also cannot be used in vivo. Solid-state photon counting image sensor technologies, developed over the last 16 years, address some of these issues.

The electron-multiplying CCD (EMCCD) was first demonstrated in 2001 [20], and has recently achieved 0.45 e^−^ RN [21]. However, dark current is amplified through the electron multiplication process, and therefore external cooling is employed [22]. The first solid-state CIS pixel array with DSERN appeared in 2015, achieving best-case 0.22 e^−^ RN in a remarkable 1.4 µm pixel pitch (PP) with 403 µV/e^−^ CVF [10]. Later, the first photon-counting CMOS imager achieved 0.27 e^−^ RN, by external cooling and a high CVF of 220 µV/e^−^ was realised by removing the reset transistor [23]. Oversampling ADCs have been employed in CIS to reduce all sources of readout noise (1/*f*, systematic temporal, source follower thermal, etc.) by correlated multiple sampling (CMS). The lowest published CIS RN in voltage (estimated by the author as CVF multiplied by RN) through four sample CMS is 31.7 µV RMS [24]. Therefore, with CVF surpassing 400 µV/e^−^ and RN as low as 31.7 µV RMS, CIS with sub 0.15 e^−^ RN appears not an unreasonable assumption in the near future.

Single photon avalanche diode (SPAD) image sensors emerged in 2002 with bump-bonded SPADs [25] onto a digital counter or time-to-digital converter (TDC) per SPAD device recording the time of arrival of single photons. High temporal resolution (≈50 ps [26]) permits time resolved imaging such as capturing light-in-flight [27], and seeing round corners [28]. These time correlated single photon counting (TCSPC) sensors have favoured the temporal precision of the photon’s arrival over spatial resolution (>44 µm) and fill-factor (<4%) which has, so far, restricted the wider adoption of these sensors. The digital circuit providing photon counting or timing occupies the majority of the pixel area to the detriment of photon detection. Chip stacking technology and the use of advanced digital CMOS process technologies are two methods that pitch reduction and fill factor increase will be achieved for SPAD-based image sensors in the future. Regardless of the technology, to realise high fill factor SPAD pixels, a trade-off is made between optical efficiency versus in-pixel functionality or the number of in-pixel transistors; low-transistor count analogue circuits will always be more compact than digital circuits. Our recent research has focused on time resolved photon counting applications using alternative analogue pixel designs that achieve higher fill factor and smaller pixel pitch, namely analogue counters [15], time-to-amplitude converters (TAC) [29,30] and single bit binary memories [17].

Binary SPAD-based imagers, with the capability of recording one SPAD avalanche within an integration time, were first published in 2011 [31] and have recently been published at 65 k binary pixels [32] and in our work at 77 k binary pixels [17]. Binary black and white imaging is not inherently practical for many imaging applications, therefore spatio-temporal oversampling is employed to create gray levels [14,19]. SPAD-based image sensors based on analogue counting techniques first appeared in [33] and have recently been demonstrated with 8 to 15 µm PP commensurate with CCD, EMCCD and sCMOS image sensors, and fill factor (FF) as high as 26.8% [15,16,34]. These sensors achieve time-gating comparable to gated photo-cathodes in the nanosecond [18] and sub nanosecond range [34]. Analogue-based SPAD imagers employ conventional CIS readout techniques and so, to aid comparison with CCD and CIS, equivalent metrics may be applied such as:

- Sensitivity, of the counter circuit to one SPAD avalanche event in mV/SPAD event, equivalent to CVF (or CG).
- Maximum number of SPAD events equivalent to full well.
- Input referred RN normalising voltage RMS RN to one SPAD event instead of one photoelectron.

These equivalencies are used throughout this paper. SPAD-based image sensors are the first solid-state imaging technology to have demonstrated sub 0.15 e^−^ RN, and as such provide a look-ahead to the signal and noise characteristics of DSERN image sensors in CMOS and other technologies.

## 3. Single Photon Counting Noise Modelling and Analysis

The first part of this section details a model of read noise and sensitivity (or CVF) developed to characterise our recent work in SPAD-based imaging. The second part discusses three noise measurement methods for DSERN image sensors based on the photon counting histogram (PCH). The use of single photon counting histograms are not new to the imaging community but the analysis presented here seeks to model and quantify the noise measurements that may be obtained from the PCH. A discrete Poisson probability density function (PDF) may represent photoelectrons (or photons) either from multiple reads of a single pixel or a single read of multiple pixels. For a single pixel “*i*”, the PDF for the captured photoelectrons k may be represented as:

(1) $$P\left(i,k\right)=\frac{\lambda^{k}exp\left(-\lambda \right)}{k!}:k\in \mathbb{Z}$$

where λ = mean number of photoelectrons in the integration period. PRNU may be modelled to first order as a normal distribution with mean CVF µ*_CVF_* and variance σ*_CVF_^2^*. For each electron *k*, the ideal voltage domain input signal *S_IN_* is created with the signal from each electrons at a separation *v*_(*i*,*k*)_ equal to the CVF for that pixel “*i*”:

(2) v(i,k)=k·CVF(i)

(3) $$S_{IN}\left(v_{k,i}\right)=P\left(i,k\right)$$

For each electron *k*, assuming the read noise is dominated by thermal noise it follows a Gaussian distribution. Read noise σ*_RN_* is applied on each electron’s output signal *S_k_* for the range *v* = 0 to (*n*.CVF): where *n* is the maximum number of electrons in the Poissonian PDF in Equation (1):

(4) $$S_{k}\left(v\right)=\frac{1}{\sigma_{RN}\sqrt{2\pi }}\cdot exp\left(-\frac{{\left(v-S_{IN}\left(v_{k}\right)\right)}^{2}}{2{\sigma_{RN}}^{2}}\right)$$

The voltage domain output signal is then represented as the summation of each of the constituent signals for each electron within the PDF:

(5) $$S_{OUT}\left(v\right)=\overset{n}{\underset{k=0}{\sum }}S_{k}\left(v\right)$$

**Figure 1:**
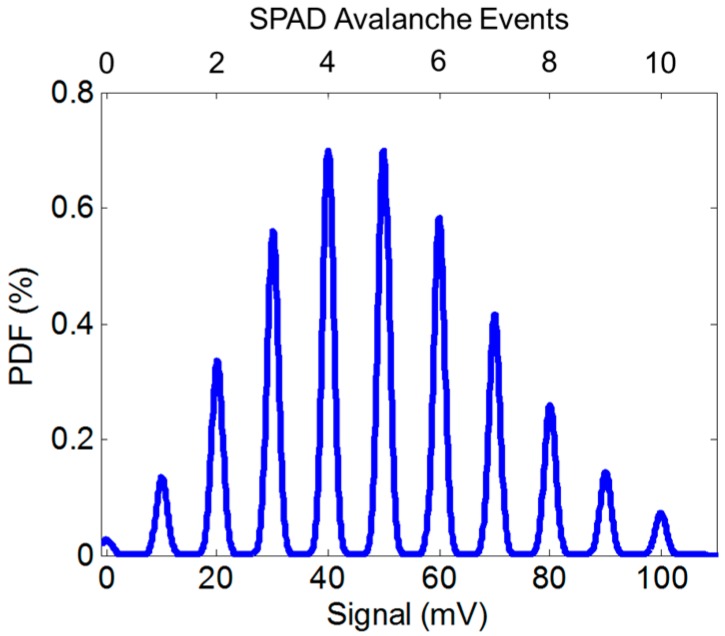
Photon counting histogram (PCH) generated by the read noise model with CVF equivalent of 10 mV/e, mean λ = 5 e^−^ exposure and 0.1 e^−^ equivalent RN.

Figure 1 provides a photon counting histogram (PCH) example of the output of the model given by Equation (5) with 10 mV/SPAD event (or 10 mV/e^−^ equivalent) and 0.1 e^−^ equivalent RN. As seen in the figure, discrete peaks are visible in the PCH. The RN distribution around each photon counting peak can be determined using three recent methods: 

### 3.1. Valley to Peak Ratio Method

Fossum et al. proposed the Valley to Peak ratio Method (VPM) detailed in [10,35]. This measures the peak height and the neighbouring valley height (or dip between photon peaks) in the PCH. The VPM has an upper and lower RN measurement limit. Although theoretically possible, it is difficult in practice to obtain peaks and valleys in PCHs in the region of 0.5 e^−^ to ≈0.45 e^−^ RN giving an upper limit to VPM. At the lower limit, below 0.15 e^−^ RN, the VPM is inherently restricted as the valley has reached the “floor” of the PCH (zero counts in more than one adjacent bin), and a companion method is needed.

### 3.2. Peak Separation and Width Method

The Peak Separation and Width (PSW) method is proposed in this paper, and has been used in this paper to measure the SPAD-based image sensors in our recent work [15,16]. The previous VPM measurement evaluates vertically in the PCH, whereas this PSW method operates in the voltage domain or horizontally in the PCH. By determining, the centroid of each single photon counting peak (whether by taking the peak position, or using a centroid weight algorithm, or similar), the peak separation data may provide a number of measurements:

- The sensitivity or CVF per pixel (“*i*”) is established by mean peak separation in a per-pixel PCH.
- The PRNU and the average CVF of the sensor are evaluated through a histogram of the compiled peak separation data from step 1 above, taking RMS and mean respectively.
- Vertical, horizontal and pixel to pixel FPN (VPFN, HFPN, PPFPN) are exhibited as horizontal offsets to the peaks, in the set of per pixel PCHs.

The width of each peak is measured to deduce the noise characteristics of the sensor. The full width half maximum (FWHM) of each peak is captured (preferably using interpolative fitting between PCH bins to lessen errors from quantisation and non-linearity in calculations). Assuming the noise around each peak is normally distributed, the FWHM may be converted to standard deviation using the conventional expression:

(6) $$\sigma =\frac{FWHM}{2\sqrt{2\ln2}}\to \sigma \approx \frac{FWHM}{2.3548}$$

The interested reader may create a more complete noise model by expanding Equations (4) and (6) to take into account other read noise sources (reset, flicker, etc.). Ideally the peak width remains constant across the full signal range, and RN is determined by the mean of the peak width data. However, if a signal dependent noise source is present then the peak widths will increase (and peak heights decrease) for increasing signal. There is no lower limit to the PSW method. However, the upper limit is set by the height of the valley between two peaks: by definition this valley must be lower than half of the two adjacent peak heights which evaluates at <0.3 e^−^ RN approximately.

### 3.3. Regressive Modelling and Fitting Method

The third method fits and scales the noise model described above, against a PCH (whether a single exposures of a full sensor or multiple exposures per pixel). This method has been used in [23] to graphically confirm the correct evaluation of RN and mean exposure. This method is expanded here to encompass the previous two methods. First the VPM and PSW are used (as appropriate given their respective limits) to obtain an estimate of RN and CVF to restrict the scaling and fitting “search” domain. Next the iterative process begins, recording the goodness of fit of the recorded PCH to the modelled PCH and continuing the regression analysis (by whichever chosen fitting method). 

Like the PSW method, this regression analysis should be performed per pixel to obtain the CVF, PRNU and FPN distributions of the image sensor. Furthermore, as in PSW, ADC non-linearity will affect the regression analysis so some method of interpolation between PCH bins may be necessary. The downside to this method, is its computationally intensive nature and the requirement to have a consistent mean number of photons for exact fitting. The Poisson distribution in Equation (1) assumes a constant mean number of photoelectrons (i.e., constant light level) through successive reads of a single pixel, and a constant light level across the array with equal sensitivity (0% PRNU). The advantage of the method is that the model can be expanded to account for known converter non-linearity or other noise sources, such as described in the following sections.

## 4. Analogue Counter and Photon Counting Performance

Single photon counting is achieved in the analogue domain with a SPAD avalanche pulse triggering an integrator circuit based on the principle of the charge transfer amplifier (CTA) whose operation is briefly described here, and in further detail in [15,16]. 

**Figure 2:**
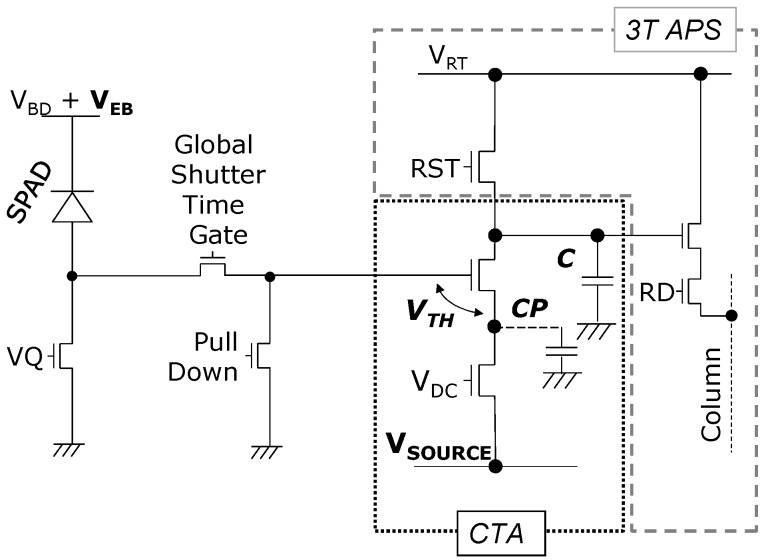
Charge transfer amplifier (CTA) analogue integrator pixel with active pixel sensor (APS) readout for global shutter or time-gated SPAD-based photon counting imaging.

In reference to Figure 2, the SPAD is connected to a passive quench and recharge transistor with static DC bias voltage “*V_Q_*” controlling the recharge or “dead” time of the diode. This is connected to the analogue counting CTA circuit via a two transistor global shutter time gate. The CTA is reset by pulling the main capacitor “*C*” to the high reset voltage *V_RT_*. The CTA operates by the input gate voltage (in this case the SPAD anode voltage) increasing above the threshold voltage of the input source follower. Charge flows from the main capacitor “*C*” to the parasitic capacitor “*C_P_*” and the voltage rises on the parasitic node. The rising voltage pushes the source follower into the cut-off region and the charge flow halts, causing a discrete charge packet to be transferred from the main capacitor for each input pulse. The SPAD anode begins recharging and the lower transistor in the CTA discharges the parasitic capacitance which is achieved with a static bias voltage “*V_DC_*” applied keeping this transistor, below threshold, in weak inversion.

The voltage step sensitivity (CVF equivalent) of CTA pixels is determined by the fixed capacitor ratio (parasitic capacitance “*C_P_*” divided by integration capacitor “*C*”) scaling down the input voltage spike. The CTA voltage step (“$\Delta V_{CTA}$”) is bias controllable by “*V_SOURCE_*” and given to a first order by the equation:

(7) $$\Delta V_{CTA}=\left(\frac{C_{P}}{C}\right)\;\cdot \;\left(V_{EB}-V_{SOURCE}-V_{TH}\right)$$

where *V_EB_* is the excess bias of the SPAD above the breakdown voltage *V_BD_*, *V_SOURCE_* is the global CTA source bias voltage, and *V_TH_* is the threshold voltage of the CTA input transistor.

**Figure 3:**
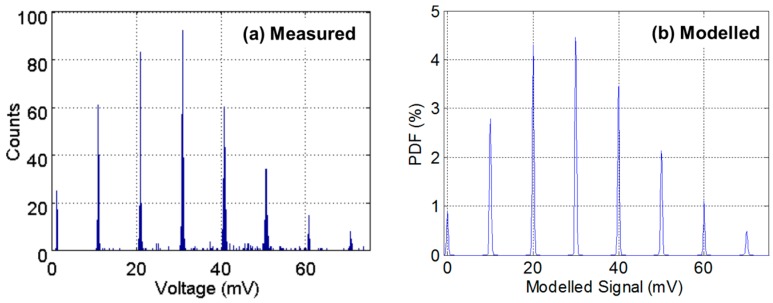
(**a**) Measured PCH of the analogue counting pixel test structure in [15]; (**b**) Modelled PCH with mean λ = 3 SPAD events, CVF equivalent of 10 mV/SPAD event and equivalent 0.02 e^−^ RN.

Figure 3a illustrates an example of the output of one test structure pixel recorded with 1000 repetitions of 30 μs integration time and ADC conversion from [15]. 1000 repetitions were chosen to give an adequate number of data samples versus experimental time. The SPAD is biased at 2.7 V *V_EB_* above breakdown voltage *V_BD_* ≈ 13.4 V. The discrete peaks under a classical Poisson distribution are clearly evident indicating the photon counting in this example is shot noise limited. Figure 3b is the side-by-side modelled PCH from a manual regressive modelling and fitting method analysis. The parameters were chosen for the closest found fit, although an offset in the *x*-axis is still present. In Figure 3a, there is a slight “in-filling” of some data values between the peaks. This is attributed to a distortion mechanism in the passively operated CTA circuit due to the imperfect reset, or incomplete discharge, of the parasitic capacitance *C_P_* for short inter-arrival times of two SPAD avalanche events less than 100 ns apart. 

**Figure 4:**
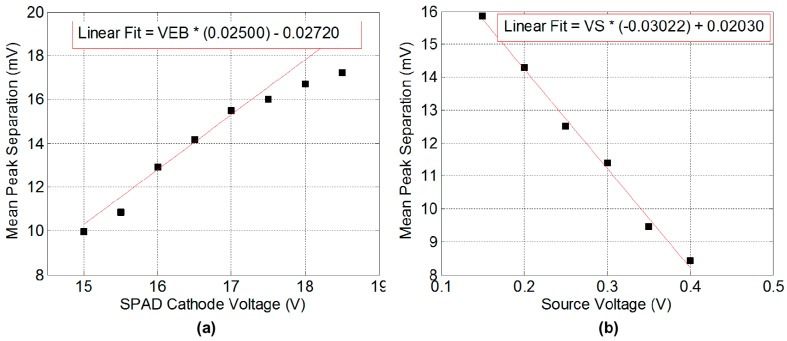
Measured mean peak separation from a set of PCHs, (**a**) The relationship of counter sensitivity to SPAD operating voltage; (**b**) The relationship to CTA *V_SOURCE_* voltage.

The PSW method is performed for the image sensor in [16] to determine the response of the analogue counter to the SPAD excess bias and the source bias voltage. Figure 4 illustrates the relationship of the mean peak separation or image sensor sensitivity to both the SPAD excess bias and the CTA source voltage. The absolute value of the linear gradient fitting parameter indicates the capacitor ratio whilst the offset parameter indicates the other terms in the CTA equation.

The linear full well (defined as a deviation of 3% in sensor output from an ideal linear response) is measured against the CTA *V_SOURCE_* bias, and the data are presented in Table 1. This demonstrates the trade-off of increasing full well against lower sensitivity and increasing RN.

## 5. Analogue Counter Cumulative Noise

Through noise measurement and iterative modelling, it is established that the analogue integrator circuits employed in SPAD-based counting pixels suffer from cumulative noise. For each SPAD event, noise affecting the counter circuit modulates the circuit sensitivity, and as the pixel integrates, the noise cumulates. Although the passive CTA pixel suffers from the “in-filling” distortion mechanism described in the previous section, all analogue integrator structures such as CTAs or switched current sources (SCS) [36,37] circuits will suffer from cumulative noise to a certain degree. The two main sources of cumulative noise are thermal noise through the switched path (which exhibits as a kT/C noise on the in-pixel capacitor, with the SPAD dead time, or counter switch time, controlling the thermal noise bandwidth) and systematic temporal noise on the common supplies. Of course, for long integration times, 1/*f* noise in the counter circuit and low frequency temporal noise on the common supplies will also modulate the integrator sensitivity and contribute cumulative noise. 

**Figure 5:**
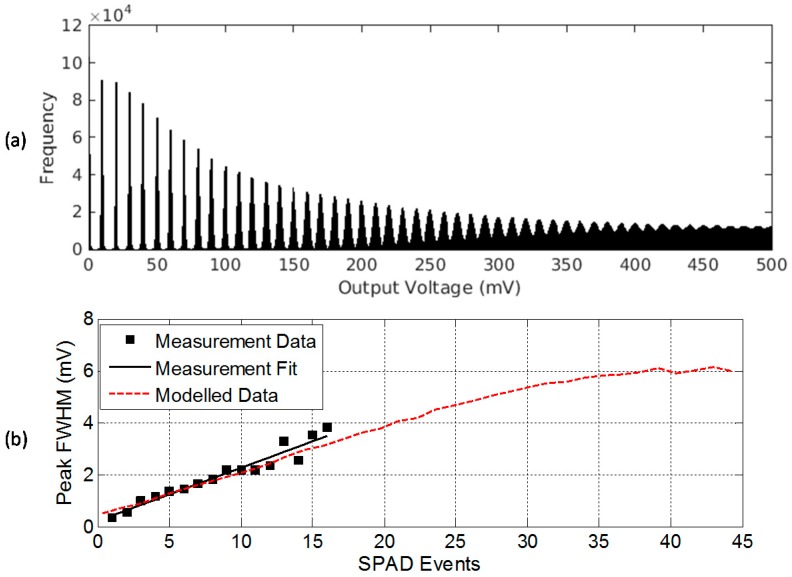
(**a**) Modelled multiple exposure PCH of a signal dependent cumulative noise source in the SPAD-based analogue counter structure [15]; (**b**) Measured and modelled peak FHWM, the first order linear fit has parameters: offset 204.7 µV with cumulative noise FWHM 225.9 µV/SPAD event = 86.9 µV/SPAD event RMS. The modelled data has cumulative noise 86.9 µV/SPAD event applied.

The PSW method is employed on one pixel in the test array in [15] to evaluate for this cumulative and signal dependent noise source. Multiple experiments were captured (each with an individual PCH as seen in Figure 3), and for each experiment the integration time (from 1 µs to 100 µs) was increased to obtain greater number of SPAD events. An example of the combined PCH is modelled in Figure 5a. Figure 5b extracts the increasing peak width indicating the presence of a cumulative noise source (σ*_C_*) from measured data. A linear fit (solid black line) identifies an σ*_C_* = 86.9 µV RMS noise increase per SPAD event. The model shown in Figure 5a is matched with 86.9 µV RMS noise per counter step and the modelled FWHM response is shown alongside (dashed red line) in Figure 5b.

The cumulative noise modelled response *S_N_* after *N* steps can be modelled to first order by expanding Equation (4) into an iterative expression assuming the cumulative noise is Gaussian. The initial reset level *S*_0_ (*N* = 0) is assumed constant with no FPN and no noise terms (a Dirac function). The first modelled counter step *S*_1_ has σ*_C_* cumulative noise applied. The second step *S*_2_ is the convolved response of the first counter step with the same Gaussian cumulative noise, and so on, as an iterative convolution for subsequent counter steps as shown in Equation (8):

(8) $$S_{N}\left(v_{N}\right)=\frac{1}{\sigma_{C}\sqrt{2\pi }}\cdot exp\left(-\frac{{\left(v_{N}-S_{N-1}\left(v_{N-1}\right)\right)}^{2}}{2{\sigma_{C}}^{2}}\right)$$

where the *v_N_* represents the voltage range of interest.

The same PSW procedure is performed for the full 320 × 240 image sensor in [16]. The imager has 700 µV RMS noise per SPAD event, an increase of approximately 8 times. This is attributed to an increase of kT/C noise due to both the main and parasitic capacitors decreasing in size between the sensors, the capacitance ratio increasing from approximately doubling from 0.013 to 0.03, and an increase in temporal noise due to many more pixels active on the same supplies. Although it is noted, that some fraction of the increase may also be attributed to ≈1% PRNU which would manifest similarly with a ≈100 µV RMS broadening of the peaks per counted photon. 

**Figure 6:**
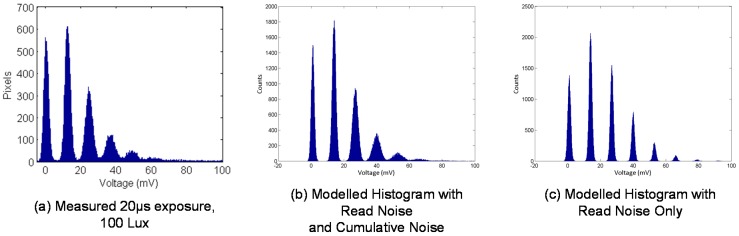
(**a**) Measured PCH for all pixels in the 320 × 240 image sensor in [16]; (**b**) Modelled PCH (mean λ = 1.5 e^−^) accounting for both cumulative noise and read noise showing close fit to the measured PCH; (**c**) Modelled PCH with read noise only showing a different response.

Figure 6a gives an example PCH from the imager. Figure 6b is the PCHs of the noise model applying 700 µV RMS cumulative noise and 0.06 e^−^ RN, and Figure 6c applying only RN. Figure 6b has a much closer fit to the captured PCH, whereas Figure 6c indicates the shape of a PCH that a CIS DSERN sensor with 0.06 e^−^ RN should achieve. With such a cumulative noise source, the equivalent input referred read noise increases depending on exposure. Table 2 presents the signal against the equivalent input referred noise figures for both the imager and test structure.

## 6. Spatio-Temporal Oversampling of Photon Counting Pixels

As analogue SPAD pixels suffer from increasing cumulative noise at higher photon counts and the effective full well is restricted, oversampling individual frames at low photon counts provides a means to create an image of high dynamic range with low overall noise. This section addresses trade-offs, and details different methods, of spatio-temporal oversampling of photon counting pixels. The Quanta Image Sensor (QIS) framework proposed by Fossum [38], extrapolates the imaging trends of pixel shrink, increasing CVF, decreasing RN, decreasing full well and spatio-temporal oversampling to a concept of a SPC image sensor where a “pixel” is the spatio-temporal sum of multiple integrations of multiple sub-pixels (“jots”).

The small full well of photon counting pixels, in the order of magnitude of 100’s of photoelectrons or photons, limits a sensor’s dynamic range. Spatio-temporal oversampling of multiple pixels may be performed to increase the full well past a single pixel’s limit. Furthermore, for DSERN photon counting pixels with cumulative noise such as the SPAD-based analogue pixels described in this paper, the level of the photon counting oversampling threshold (i.e., if pixel output >1 photon or if >2 photons, etc.) sets the noise of the oversampled output image; a higher oversampling threshold induces greater noise in the output frame image. However, this threshold is traded off against the frame rate and the oversampled full well. A signal level of N photoelectrons can be reached with less oversampled frames (and greater output frame rate) with a higher oversampling threshold of the pixel signal. By setting the threshold above the thermal and 1/*f* noise floor, the oversampled is truly shot noise limited as little or no thermal and 1/*f* noise accumulates.

### 6.1. Single Photon Binary Quanta Imaging

**Figure 7:**
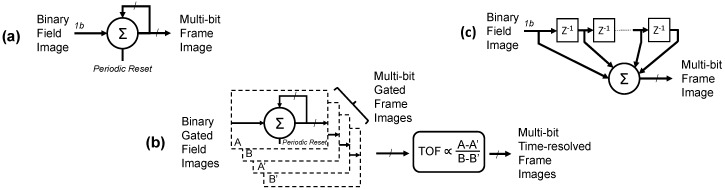
Per-pixel spatio-temporal oversampling techniques. (**a**) Intensity image using IIR with periodic reset [17]; (**b**) Time-resolved image: four IIR per pixel [18]; (**c**) High frame rate intensity image using first-order FIR per pixel [19].

Using SPAD-based single photon image sensors with binary response, a variety of oversampling techniques have been evaluated in our recent work [16,17,18,19] and in the work of others [14,39]. “Field” images are individual reads from the image sensor and the oversampled frame is a summation of fields. The simplest technique in order to oversample a set of binary single photon field images, is to temporally or spatially sum a set of input binary pixel (or “jot”) values, to create an output “macro” pixel with grey levels. This is the equivalent operation of a first-order low-pass infinite impulse response (IIR) filter with a periodic filter reset operation as shown in Figure 7a. Considering temporal oversampling only (as demonstrated in [17]), to achieve a certain output frame rate in FPS, with oversampled ratio OSR and input binary field rate f, the output rate is: FPS = *f*/OSR and inversely the IIR reset period = OSR/*f*, thus attaining an output bit depth of B = Log2(OSR), increasing the image bit depth by a factor of OSR or 2^B^. It is clear that to attain frame rates >30 FPS, at bit depths B > 5 bit, a high field rate *f* > 1 k fields/s is required from the sensor. In [17], we demonstrated 7b bit depth at 40 FPS, and 8b at 20 FPS with 5.12 k global shutter fields per second.

SPADs with picosecond temporal precision enable Indirect Time of Flight (ITOF) imaging to be performed. Previous examples are pulsed ITOF using analogue pixels [33] and continuous wave ITOF using digital pixels [40]. However, both approaches had very large pixel pitch and low fill factor. A similar oversampling technique was applied in [18] with compact binary SPAD pixels, to investigate time-gated binary image oversampling to produce a high resolution QVGA Indirect Time of Flight (ITOF) output image as shown in Figure 7b. Two primary gated field images (A & B) are sequentially captured in interleaved fashion synchronous to a pulsed laser. Two secondary gated images (A’ & B’) are set with the same time-gate without the laser for background removal. With four field images, the output time-resolved frame rate is therefore a quarter of the previous intensity-only technique (assuming a pipelined division operation).

A third technique in [19], addresses the low frame rate, and evaluates a continuous-time moving average operation by applying a first-order low-pass finite impulse response (FIR) filter. As shown in Figure 7c, the FIR is implemented as a shift-register of length equal to the over-sampling ratio (i.e., a FIR with number of taps = OSR) and a tracking counter. The benefit of this technique is the output frame rate has no relationship with the OSR and is equal to the input field rate of the sensor. The frame rate increase over the IIR technique is at the cost of the shift register per pixel. Longer integration time increases temporal blur, therefore, higher OSR increases image lag of fast moving scene elements. On the other hand, an increased bit depth (from greater OSR) decreases quantisation noise in areas of slow movement in an imaged scene.

We compare our recent work in this area, to two others demonstrating high binary field rates with column parallel single bit flash ADCs for single bit QIS in Table 3. In a 3T CIS implementation [41], amplification and CDS is employed and suitable for pixels with low signal swing (i.e., CVF ≤ input-referred offset and read noise). In our work [16] and another SPAD-based example [42], no CDS or column amplifier circuits are required as the pixel sensitivity is >1 V/SPAD event which is much greater than offsets and RN. The RN and non-linear exposure characteristics of such oversampled binary imagers are theoretically described in [9] and experimentally confirmed in our work in [16,18]. The measured bit error rate is 0.0017 providing an equivalent DSERN of 0.168 e^−^ Without CDS timing and increased column current, the field rate more than doubles [16].

### 6.2. Multi-Photon Binary Quanta Imaging

As previously discussed, setting the oversampling threshold greater than a single counted photon provides a benefit to output frame rate assuming the sensor output data rate remains the same. By setting the oversampling threshold at two photons rather than one, half number of field readouts are required to reach a certain oversampled signal level as each binary bit now represents more than one photon. However, for the SPAD-based analogue counting pixel this is at the cost of oversampling greater cumulative noise, FPN or PRNU with each successive field image. 

**Figure 8:**
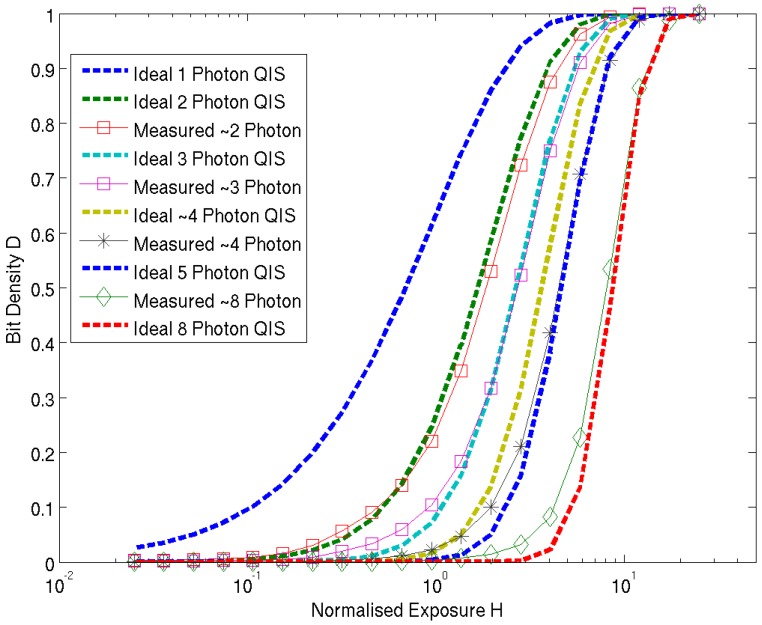
Multi-photon threshold oversampled binary imaging normalised bit density to exposure. Ideal curves from [9] are presented alongside measured results.

An experiment is performed on the image sensor [16] recording the “bit density” (the number of pixels outputting a logical high indicating the multi-photon counting threshold is reached) against increasing integration time for a fixed light level. The pixel array in configured in analogue counting CTA mode with V*_SOURCE_* = 0.15 V. Figure 8 highlights the normalized bit density (D) to normalized exposure (H), where 1.0 H = 5 µs integration time, for an incrementing comparator threshold capturing two to eight photons. The theoretical curves from [9] are plotted alongside for comparison. As no CDS is implemented, the high FPN due to column comparator mismatch and source follower threshold variation will effectively induce a PRNU in the measured data for all pixels which is seen as the discrepancy between ideal and measured data particularly in the plotted line for the four photon threshold. The closest fit in terms of photon number (2 to 8) is listed in the legend alongside. 

**Figure 9:**
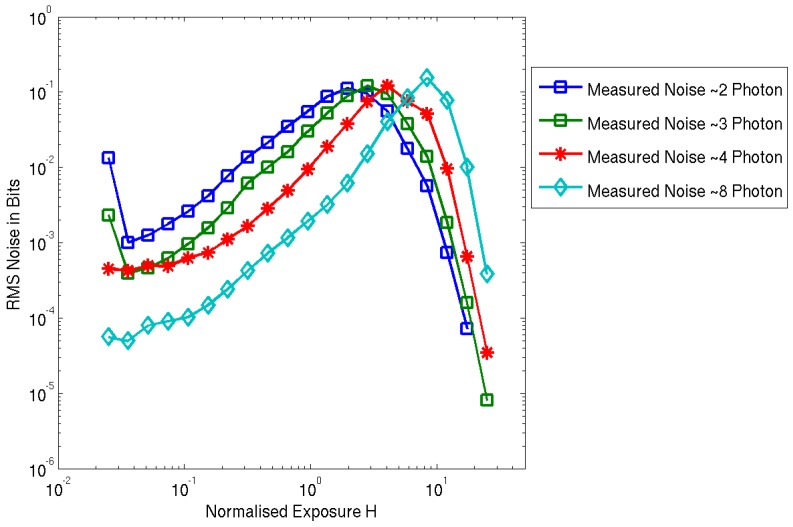
Measured RMS noise in multi-photon threshold oversampled binary imaging.

Figure 9 is the measured normalized RMS noise which has the characteristic shape from Fossum’s theoretical Quanta Image Sensor paper in [9]. A few remarkable characteristics of multi-photon threshold binary imaging that are experimentally verified in this noise plot. The rising slope of each of the noise plots indicates the shot-noise dominant region. The 2-photon line demonstrates the “soft-knee” shot noise compression with a smooth roll-off after the peak after H = 1.0 as expected in 1-photon or 2-photon threshold QIS. The subsequent increasing thresholds show a horizontal shift in the exposure *x*-axis as a higher number of photons (or equivalent SPAD events) are required to trigger the binary output. This can also be observed in the horizontal shift in the D-LogH plot in Figure 8. The maximum noise in the 8-photon threshold is measured as 1.52 times higher than the 2-photon threshold where the theory [9] suggests it should be no more than square root of two higher (1.412 times).

## 7. Discussion

Table 4 provides a comparison table highlighting a selection of state of the art solid-state photon counting image sensors in the three different technologies (CIS, EMCCD and SPAD). This section discusses and compares the performance of SPAD-based image sensors based on analogue integration. SPAD based image sensors have the highest CVF of solid-state SPC image sensors. Moreover, the pixel size of the SPAD analogue-based imagers is commensurate with EMCCD and sCMOS scientific imagers, although FF is lower. With the exception of the LOFIC pixel which has dual CVF’s, like the recent CIS DSERN pixels, the increase in CVF of SPAD pixels yields a reduced full well in the order of 100’s photo-electrons or integrated SPAD events.

SPADs have the advantage of picosecond temporal resolution. Analogue pixels with low transistor counts permit nanosecond and sub-nanosecond time-gated SPC imaging to be realized where digital pixels further permit TCSPC imaging with 10’s ps time resolution at the cost of low spatial resolution.

In terms of RN, SPAD analogue integrators share a similar noise characteristic with 3 transistor (3T) CIS pixels in that the integration node is not fully depleted and so suffers from kT/C noise. Our test structure [15] cancels the kT/C noise by implementing 3T-pixel true CDS timing and furthermore implemented 4096 sample CMS to yield <0.01 e^−^ equivalent RN in the best case. However, both 3T timing and >1 k sample CMS is very restrictive in an image sensor design preventing, for example, the global shutter or global time-gated operation that our recent work and [37] implements. Therefore delta-reset sampling CDS [43] is implemented in our SPAD analogue counter image sensor which adds a noise component of 100’s µV RMS kT/C to the RN. However, the equivalent CVF of the SPAD-based analogue pixels in the 10 mV range is high enough to compensate, as demonstrated by the 0.06 e^−^ RN figure which is the lowest in the published SPC image sensor literature.

In comparison to other works, analogue integrators suffer from cumulative noise limiting the photon number resolution. Spatio-temporal oversampling, at a few photons per pixel level, mitigates the noise integration whilst extending the photon number resolution although high frame rates are required.

## 8. Conclusions

Our recent work on SPAD-based photon counting image sensors is analysed for photon counting performance and deep sub electron equivalent noise characteristics. A noise model is developed to include both CIS RN and the cumulative noise specific to analogue integrator circuits. When combined, the three new methods (VPM, PSW and regressive analysis) of determining RN form a new powerful set of tools for the measurement of most SPC and DSERN image sensor characteristics alongside the existing techniques such as photon transfer curve analysis.

These single-photon and multi-photon methods of binary image capture have the attractive quality of similar noise and signal characteristics of photographic film. Future development of these binary photon-counting image sensors is an interesting and new avenue of research. The tradeoff between in-pixel cumulative and spatio-temporal oversampling is examined. Analogue SPC pixels have DSERN but exhibit cumulative noise limiting photon number resolution. As a result they are best operated at low photon number in combination with digital oversampling. A very large dynamic range is conceivably possible, combining the multi-photon counting with an oversampled frame store, which would extend the limited dynamic range of the analogue counter. Furthermore, the frame rate penalty of oversampling is addressed by a continuous-time moving average technique.

The capability of an image sensor to capture the arrival of a single photon, is the fundamental limit to the detection of quantised electromagnetic radiation. Each of the three solid-state SPC image sensor technologies, CMOS SPAD, EMCCD and DSERN CIS have specific advantages that will individually serve a variety of photon counting applications.

## Figures and Tables

**Table 1:** Photon counting performance of 320 × 240 SPAD-based image sensor [16].

*V_SOURCE_* Bias Voltage (mV)	Linear Full Well Voltage (mV)	Sensitivity from Linear Fit (mV/SPAD Event)	Input Referred Read Noise (SPAD Events)	Equivalent Linear Full Well (SPAD Events)
200	802.8	14.26	0.064	56
300	722.1	11.23	0.082	64
400	651.4	8.21	0.113	79
500	648.3	5.19	0.178	125

**Table 2:** Equivalent noise at a range of SPAD events.

Equivalent Input Referred Total Noise	No. of SPAD Avalanche Events	
	Image Sensor [16]	Test Structure [15]
0.15 e^−^	2	19
0.3 e^−^	5	45
1 e^−^	19	160

**Table 3:** Binary capture, oversampled output, quanta image sensor comparison table. FOM† = Sensor power/(No. of Pixel × FPS × N), where N = ADC resolution = 1b for these sensors.

Reference	[41]	[42]	[16,17]
**Sensor Name**	QIS Pathfinder	SwissSPAD	SPC Imager
**Process Technology**	180 nm CMOS	0.35 µm HV CMOS	130 nm Imaging CMOS
**Array Size**	1376 × 768	512 × 128	320 × 240
**Photo-detector**	“Pump-gate Jot” PD	SPAD	SPAD
**NMOS Pixel Transistors**	3	11	9
**Fill Factor (%)**	45	5	26.8
**Pixel Pitch (µm)**	3.6	24	8
**Microlensing**	N	Y (12× concentration factor)	N
**Shuttering**	Rolling	Global	Global
**CDS**	True CDS	None	None
**Parallel Data Channels**	32	128	16
**Max. Field Rate (FPS)**	1000	150,000	20,000
**Sensor Data Rate**	1 Gbps	10.24 Gbps	1.54 Gbps
**Pixel CVF or Equivalent**	120 µV/e^−^	>1 V per SPAD Event	>1 V per SPAD Event
**Bit Error Rate**	Not Reported	Not Reported	1.7 × 10^−3^ BER
**Read Noise (e^−^) or Equivalent**	Not Reported	Not Reported	0.168 e^−^
**Power During Operation**	20 mW	1650 mW	40.8 mW
**Power FOM†**	2.5 pJ/b (ADC only) 19 pJ/b (Full Sensor)	168 pJ/b (Full Sensor + SPADs)	104 pJ/b (Full Sensor + SPADs)

**Table 4:** Solid state single photon counting image sensor comparison table.

Reference	[12]	[10]	[23]	[21]	[37]	[15]	[16]	[26]
**Photodetector**	PIN PD + LOFIC	“Pump-gate Jot” PIN PD	PIN PD	EMCCD	SPAD	SPAD	SPAD	SPAD
**Pixel Circuit**	5T + LOFIC	4T	4T	CCD	Active CTA 8T	Passive CTA 11T	Passive CTA 9T	7b Counter >100T
**Array Size**	1280 × 960	1	35 × 512	1920 × 1080	160 × 120	3 × 3	320 × 240	32 × 32
**Pixel Size (µm)**	5.6	1.4	11.2 × 5.6	5.5	15	9.8	8	50
**Fill Factor (%)**	30.4	-	-	50	21	3.12	26.8	1
**Pixel CVF or Equivalent**	240 µV/e^−^	403 µV/e^−^	220	Gain dependent from 44 µV/e^−^	16.5 mV/SPAD event	13.1 to 2 mV/SPAD Event	17.4 to 8.4 mV/SPAD event	1 DN/SPAD Event
**Full Well or Equivalent**	200 ke^−^	210 e^−^	1500 e^−^	20 ke^−^ to 160 e^−^	41	80 to 360	56 to 125	127
**Read Noise (or Equivalent)**	0.41 e^−^	0.22 e^−^	0.27 e^−^	0.45 e^−^	0.08 e^−^	<0.01 e^−^ to 0.22 e^−^	0.06 e^−^ to 0.18 e^−^	0
**Excess Noise**	-	-	-	Y	-	-	-	-
**Cumulative Noise**	-	-	-	-	Y *	Y	Y	-
**Measured Cumulative Noise**	-	-	-	-	Not Measured	86.9 µV RMS/SPAD Event	700 µV RMS/SPAD Event	-
**Time Gating Width or Temporal Resolution**	-	-	-	-	0.75 ns	100 ns	30 ns	52 ps

* As based on a CTA analogue integrator structure, the presence of cumulative noise is assumed by the author.

## Abbreviations

The following abbreviations are used in this manuscript:

CG	Conversion Gain
CIS	CMOS Image Sensor
CTA	Charge Transfer Amplifier
CVF	Charge to Voltage Conversion Factor
DSERN	Deep Sub Electron Read Noise
EMCCD	Electron Multiplied Charge Coupled Device
FIR	Finite Impulse Response Filter
IIR	Infinite Impulse Response Filter
PCH	Photon Counting Histogram
PSW	Peak Seperation and Width method
QIS	Quanta Image Sensor
RN	Read Noise
SPAD	Single Photon Avalanche Diode
SPC	Single Photon Counting
TCSPC	Time Correlated Single Photon Counting
VPM	Valley to Peak method
